# Prevalence of computer vision syndrome during the COVID-19 pandemic: a systematic review and meta-analysis

**DOI:** 10.1186/s12889-024-17636-5

**Published:** 2024-02-29

**Authors:** Darwin A. León-Figueroa, Joshuan J. Barboza, Abdelmonem Siddiq, Ranjit Sah, Mario J. Valladares-Garrido, Suraj Adhikari, Edwin Aguirre-Milachay, Sanjit Sah, Alfonso J. Rodriguez-Morales

**Affiliations:** 1 Facultad de Medicina Humana, Universidad de San Martín de Porres, Chiclayo, 15011 Peru; 2 Centro de Investigación en Atención Primaria en Salud, Universidad Peruana Cayetano Heredia, Lima, 15102 Peru; 3 Unidad de Revisiones Sistemáticas y Meta-Análisis, Universidad San Ignacio de Loyola, Juan del Corral 937. El Bosque, Trujillo, Lima Peru; 4 Faculty of Pharmacy, Mansoura University, Mansoura, 35516 Egypt; 5 Department of Microbiology, Institute of Medicine, Tribhuvan University Teaching Hospital, Kathmandu, 44600 Nepal; 6 Department of Microbiology, Dr. D. Y. Patil Medical College, Hospital and Research Centre, Dr. D. Y. Patil Vidyapeeth, Pune, 411018 Maharashtra India; 7 Department of Public Health Dentistry, Dr. D.Y. Patil Dental College and Hospital, Dr. D.Y. Patil Vidyapeeth, Pune, 411018 Maharashtra India; 8 Universidad Continental, Lima, 15046 Peru; 9 Oficina de Epidemiología, Hospital Regional Lambayeque, Chiclayo, 14012 Peru; 10 Manipal College of Medical Sciences, Pokhara, Nepal; 11 Research Scientist, Global Consortium for Public Health and Research, Datta Meghe Institute of Higher Education and Research, Jawaharlal Nehru Medical College, Wardha, 442001 India; 12 SR Sanjeevani Hospital, Kalyanpur-10, Siraha, Nepal; 13 Master of Clinical Epidemiology and Biostatistics, Universidad Cientifica del Sur, Lima, 15067 Peru; 14 Gilbert and Rose-Marie Chagoury School of Medicine, Lebanese American University, Beirut, 1102 Lebanon

**Keywords:** Computer Vision Syndrome, COVID-19, Prevalence, Systematic review, And Pandemic

## Abstract

**Background:**

Computer vision syndrome has become a significant public health problem, especially in developing countries. Therefore, this study aims to identify the prevalence of computer vision syndrome during the COVID-19 pandemic.

**Methods:**

A systematic review and meta-analysis of the literature was conducted using the databases PubMed, Scopus, Web of Science, and Embase up to February 22, 2023, using the search terms "Computer Vision Syndrome" and "COVID-19". Three authors independently performed study selection, quality assessment, and data extraction, and the Joanna Briggs Institute Meta-Analysis of Statistics Assessment and Review Instrument was used to evaluate study quality. Heterogeneity was assessed using the statistical test *I*^*2*^, and the R version 4.2.3 program was used for statistical analysis.

**Results:**

A total of 192 studies were retrieved, of which 18 were included in the final meta-analysis. The total sample included 10,337 participants from 12 countries. The combined prevalence of computer vision syndrome was 74% (95% CI: 66, 81). Subgroup analysis based on country revealed a higher prevalence of computer vision syndrome in Pakistan (99%, 95% CI: 97, 100) and a lower prevalence in Turkey (48%, 95% CI: 44, 52). In addition, subgroup analysis based on study subjects showed a prevalence of 82% (95% CI: 74, 89) for computer vision syndrome in non-students and 70% (95% CI: 60, 80) among students.

**Conclusion:**

According to the study, 74% of the participants experienced computer vision syndrome during the COVID-19 pandemic. Given this finding, it is essential to implement preventive and therapeutic measures to reduce the risk of developing computer vision syndrome and improve the quality of life of those affected.

**Trial registration:**

The protocol for this systematic review and meta-analysis was registered in the international registry of systematic reviews, the International Prospective Register of Systematic Reviews (PROSPERO), with registration number CRD42022345965.

## Introduction

Computer Vision Syndrome (CVS), or digital eye strain, is a set of visual and ocular symptoms that affect people who use electronic devices (ED) for extended periods [1]. These devices include computers, laptops, tablets, e-readers, and smartphones [2]. Symptoms related to CVS can be classified into three categories [3–5]: visual symptoms (blurry vision, visual fatigue or discomfort, and double vision), ocular symptoms (dry eyes, redness, eye fatigue, and irritation), and extraocular symptoms (headache and pain in the shoulders, neck, and back).

Recent studies have shown that there are two categories of risk factors associated with CVS [3]: those that are of personal origin (such as poor posture while sitting, the incorrect distance between the eyes and the screen, the wrong viewing angle, the presence of medical conditions, and prolonged computer exposure) [6] and those that are computer and environmental factors (which include inadequate workstations, insufficient lighting, poor contrast and resolution, the increased presence of screen glare, excessive brightness, and light imbalance between the screen and the surrounding work environment) [7, 8].

For the past two decades, CVS has become a highly relevant health problem that affects the entire population [1]. A systematic review and meta-analysis study found that the pooled prevalence of CVS is 66% [3]. In addition, the COVID-19 pandemic and the subsequent lockdowns have led to a significant increase in the use of ED worldwide. That massive increase in the use of ED has created a conducive environment for the development of CVS, which increases the risk of experiencing its associated visual, ocular, and extraocular symptoms [9].

During this global crisis, many people have experienced a significant increase in time spent on technology-related activities, such as working from home, participating in virtual meetings, and engaging in online entertainment [10, 11]. This change in screen use habits has led to an increased risk of developing CVS [12].

The rapid evolution of technology has made ED an essential part of our daily lives [13]. However, several studies have shown that prolonged use of these devices can negatively affect people's eye health, as evidenced during the COVID-19 pandemic [14, 15]. Therefore, this systematic review and meta-analysis aimed to determine the prevalence of computer vision syndrome during the COVID-19 pandemic. This will allow the development of strategies and preventive measures to protect and improve the eye health of the population.

## Materials and methods

### Study design and protocol registration

This systematic review and meta-analysis was registered on the International Prospective Register of Systematic Reviews (PROSPERO) under the registration code CRD42022345965. Available at: https://www.crd.york.ac.uk/prospero/display_record.php?RecordID=345965.

### Eligibility criteria

The prevalence of CVS was identified using published peer-reviewed articles with observational study methods (nonrandomized cohort and intervention studies). The articles covered what was published through February 22, 2023, and had no language restrictions. Editorials, letters to the editor, randomized clinical trials, narrative reviews, systematic review papers, and meeting proceedings were not reviewed.

### Data sources and search strategy

The databases PubMed, Scopus, Web of Science, and Embase were searched extensively. The search terms used were "COVID-19" and "Computer Vision Syndrome". The search strategy is available at: https://doi.org/10.6084/m9.figshare.22141715.v1. The searches were completed on February 22, 2023, and the results were independently evaluated by three different investigators (J.J.B., DALF, and R.S.).

### Study selection

A database was created from the results of electronic searches using Endnote reference management software. Duplicate articles, irrelevant titles, and abstracts were removed. Then, three investigators (DALF, A.S., and MJVG) independently evaluated the article titles and abstracts to select those that appeared to meet the inclusion criteria. Finally, three other investigators (J.J.B., R.S., and AJRM) carefully reviewed the full-text reports and assessed whether they met the inclusion criteria before deciding whether to include them. In any disagreement, a fourth investigator (AJRM) helped resolve the differences and reach a solution. A PRISMA (Preferred Reporting Items for Systematic Reviews and Meta-Analyses) diagram was used to present the study selection procedures.

### Data extraction

Three investigators (DALF, MJVG, and J.J.B.) worked independently to extract data from the selected articles and record them in an Excel spreadsheet. Essential details of the selected studies were collected, such as author, date of publication, study design, country, sample size, response rate, prevalence of computer vision syndrome, study subjects, age, and sex. If disagreements arose over the inclusion of studies, a fourth investigator (EAM) resolved them and verified that the list of publications and extracted data did not contain duplicate articles or irrelevant information.

### Quality assessment

"The Joanna Briggs Institute Meta-Analysis of Statistics Assessment and Review Instrument (JBI-MAStARI)" [16] was used to assess the quality of articles before their inclusion in the final meta-analysis. The evaluation was based on various aspects, such as the research context, outcome and explanatory variables, explicit inclusion criteria, measurement standards, subject description, and precise statistical analysis. DALF, AJRM, and JJB independently evaluated the quality of the studies. It was classified as "high", "moderate", or "low" based on their score, with more than 7 points for high quality, 4 to 6 points for moderate quality, and less than 4 points for low quality. Any discrepancies among the researchers during the quality evaluation were resolved through discussion (Table 1).

**Table 1:** Quality of the included studies

Authors	Year	Eligibility criteria	Study subjects and the setting	Exposure measured in a valid and reliable way 'gold standard'	A specified diagnosis or definition	Confounding factors	Dealing with confounding factors	Outcomes measured in a valid and reliable way	Appropriate statistical analysis	Scores (8)	Quality (high, moderate, low)
Mohan A, et al. [17]	2021	Yes	Yes	Yes	Yes	No	NA	Yes	Yes	7	High
Alabdulkader B. [18]	2021	Yes	Yes	Yes	Yes	No	NA	Yes	Yes	7	High
Zenbaba D, et al. [19]	2021	Yes	Yes	Yes	Yes	No	NA	Yes	Yes	7	High
Noreen K, et al. [20]	2021	Yes	Yes	Yes	Yes	No	NA	Yes	Yes	7	High
Wang L, et al. [21]	2021	Yes	Yes	Yes	Yes	No	NA	Yes	Yes	7	High
Zayed HAM, et al. [22]	2021	Yes	Yes	Yes	Yes	No	NA	Yes	Yes	7	High
Abuallut I, et al. [23]	2022	Yes	Yes	Yes	Yes	No	NA	Yes	Yes	7	High
Basnet A, et al. [24]	2022	Yes	Yes	Yes	Yes	No	NA	Yes	Yes	7	High
Coronel-Ocampos J, et al. [25]	2022	Yes	Yes	Yes	Yes	No	NA	Yes	Yes	7	High
Estrada Araoz EG, et al. [26]	2022	Yes	Yes	Yes	Yes	No	NA	Yes	Yes	7	High
Munsamy AJ, et al. [27]	2022	Yes	Yes	Yes	Yes	No	NA	Yes	Yes	7	High
Roy S, et al. [28]	2022	Yes	Yes	Yes	Yes	No	NA	Yes	Yes	7	High
Seresirikachorn K, et al. [13]	2022	Yes	Yes	Yes	Yes	No	NA	Yes	Yes	7	High
Uwimana A, et al. [29]	2022	Yes	Yes	Yes	Yes	No	NA	Yes	Yes	7	High
Wangsan K, et al. [30]	2022	Yes	Yes	Yes	Yes	No	NA	Yes	Yes	7	High
Agarwal R, et al. [31]	2022	Yes	Yes	Yes	Yes	No	NA	Yes	Yes	7	High
Almousa AN, et al. [32]	2022	Yes	Yes	Yes	Yes	No	NA	Yes	Yes	7	High
Demirayak B, et al. [33]	2022	Yes	Yes	Yes	Yes	No	NA	Yes	Yes	7	High

*NA* Not assessed

### Data analysis

The extracted Excel data was imported into the R program version 4.2.3 for analysis. The studies that were included were presented narratively using tables and graphs. A meta-analysis was conducted using the random-effects model to calculate the overall effect size, and the results were displayed using a forest plot. The *I*^*2*^ index was used to analyze heterogeneity, where *I*^*2*^ = 30% indicated low heterogeneity, *I*^*2*^ = 30–60% represented moderate heterogeneity, and *I*^*2*^ > 60% showed high heterogeneity. The R meta-package was utilized for conducting the meta-analyses. A visual evaluation was performed using a funnel plot and Egger's test for asymmetry to assess publication bias, but only if the number of included papers exceeded 10.

## Results

### Study selection

**Fig. 1:**
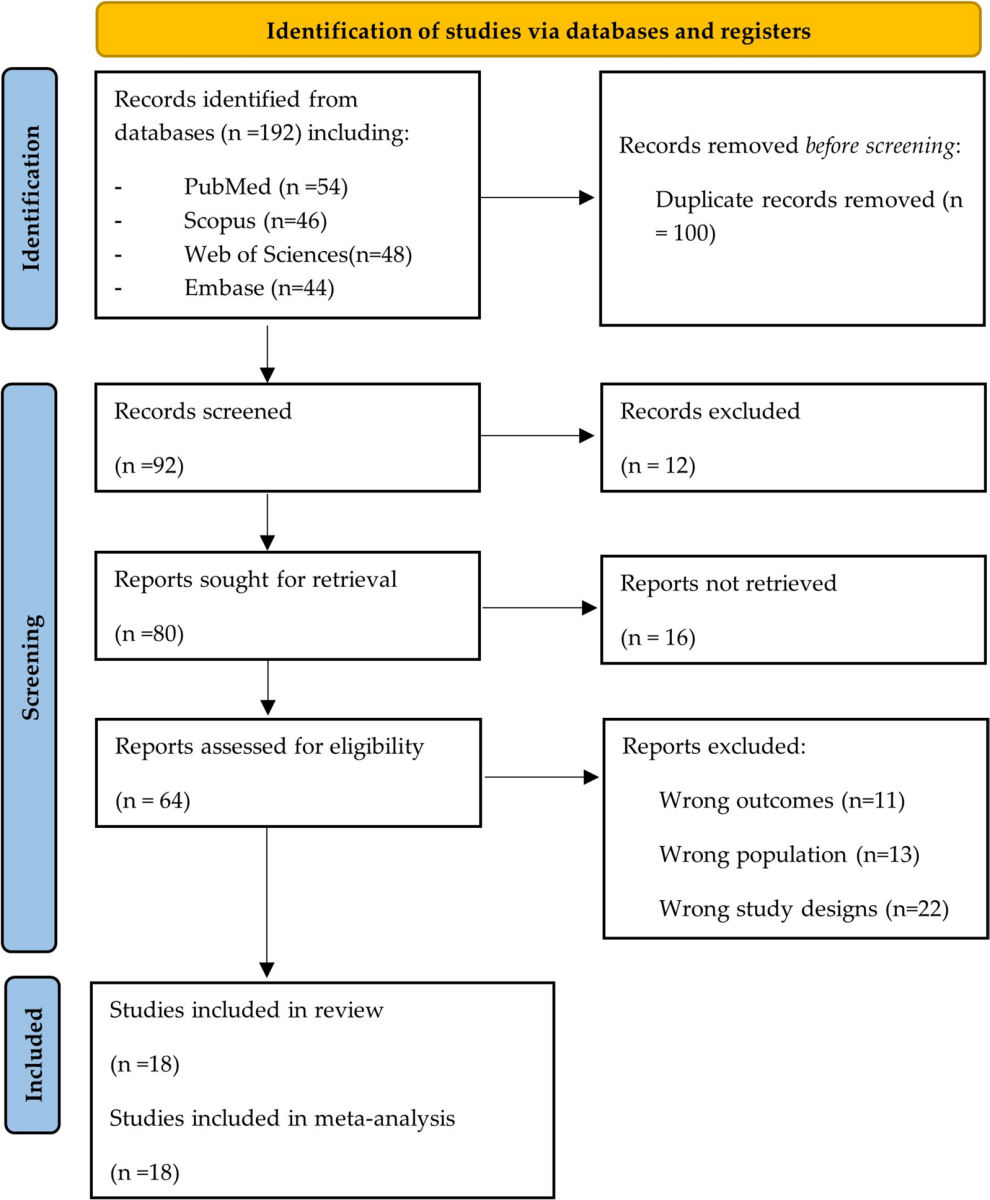
PRISMA flowchart describing the selection of studies for the systematic review and meta-analysis of the prevalence of computer vision syndrome during the COVID-19 pandemic

The search strategy allowed for the retrieval of 192 articles, whose selection process can be visualized in the PRISMA flowchart (Fig. 1). After removing duplicates (*n* = 100), 92 articles were examined by the researchers. Subsequently, after filtering the titles and abstracts, 64 articles were selected for full-text reading, and 18 were considered eligible for inclusion in this systematic review and meta-analysis [13, 17–33].

### Characteristics of the included studies

Eighteen cross-sectional studies examining the prevalence of CVS during the COVID-19 pandemic, published between 2021 and 2022, were included [13, 17–33] (Table 2). The total sample included 10,337 participants from 12 countries (*n* = 18) [13, 17–33]: India (*n* = 3) [17, 24, 31], Saudi Arabia (*n* = 3) [18, 23, 32], Ethiopia (*n* = 1) [19], Pakistan (*n* = 1) [20], China (*n* = 2) [21, 29], Egypt (*n* = 1) [22], Paraguay (*n* = 1) [25], Peru (*n* = 1) [26], South Africa (*n* = 1) [27], Bangladesh (*n* = 1) [28], Thailand (*n* = 2) [13, 30], and Turkey (*n* = 1) [33]. The sample size varied from 74 in a study of Chinese medical students at the University of Sichuan [21] to 2476 in a survey of high school students in Thailand [13]. Of the participants, 39% were men, and 61% were women. The student population was the primary participant in most included studies [13, 17, 20, 21, 23, 25–30, 32, 33]. This systematic review and meta-analysis provide a broad overview of the prevalence of CVS in various countries during the COVID-19 pandemic [13, 17–33] (Table 2).

**Table 2:** Features of research considered in a meta-analysis of computer vision syndrome during the COVID-19 pandemic

Authors	Year	Study design	Country	Sample size	Response rate (%)	Prevalence (%)	Study subjects	Age (Years)	Sex	
									**M**	F
**Mohan A, et al.** [17]	2021	Cross-sectional	India	217	83.14%	50.23%	Students	Mean: 13 ± 2.45	101	116
**Alabdulkader B.** [18]	2021	Cross-sectional	Saudi Arabia	1939	96%	78%	Population > 18 years	Range (18–81)	537	1402
**Zenbaba D, et al.** [19]	2021	Cross-sectional	Ethiopia	416	98.60%	70.40%	University instructors	≥ 24	301	115
**Noreen K, et al.** [20]	2021	Cross-sectional	Pakistan	326	95.04%	98.70%	Students	Range (17–25)	98	228
**Wang L, et al.** [21]	2021	Cross-sectional	China	74	84.09%	74.32%	Students	Range (18–20)	39	35
**Zayed HAM, et al.** [22]	2021	Cross-sectional	Egypt	108	98.18%	82.41%	Technology professionals	Mean: 32.2 ± 5.97	40	68
**Abuallut I, et al.** [23]	2022	Cross-sectional	Saudi Arabia	407	92.50%	35.40%	Students	Range (6–18)	198	209
**Basnet A, et al.** [24]	2022	Cross-sectional	India	318	98.70%	94.30%	Population > 20 years	Mean: 36	145	173
**Coronel-Ocampos J, et al.** [25]	2022	Cross-sectional	Paraguay	228	100%	82.50%	Students	Mean: 22.3	65	163
**Estrada Araoz EG, et al.** [26]	2022	Cross-sectional	Peru	215	100%	72.10%	Students	Range (16– > 31)	97	118
**Munsamy AJ, et al.** [27]	2022	Cross-sectional	South Africa	290	97.64%	64.24%	Students	Mean: 21.04	214	76
**Roy S, et al.** [28]	2022	Cross-sectional	Bangladesh	917	100%	68.16%	Students	Range (14–46)	220	697
**Seresirikachorn K, et al.** [13]	2022	Cross-sectional	Thailand	2476	100%	70.10%	Students	Range (10–19)	869	1607
**Uwimana A, et al.** [29]	2022	Cross-sectional	China	452	90.80%	50%	Students	Mean: 27.25 ± 5.62	298	154
**Wangsan K, et al.** [30]	2022	Cross-sectional	Thailand	527	100%	81.02%	Students	Mean: 20.04	156	371
**Agarwal R, et al.** [31]	2022	Cross-sectional	India	435	79.09%	81.37%	Population > 18 years	Mean: 35	224	211
**Almousa AN, et al.** [32]	2022	Cross-sectional	Saudi Arabia	300	80%	94%	Students	Mean: 21.5 ± 1.9	124	176
**Demirayak B, et al.** [33]	2022	Cross-sectional	Turkey	692	97.19%	48.20%	Students	Mean: 9.72 ± 3.02	332	360

### Quality of the included studies and publication bias

**Fig. 2:**
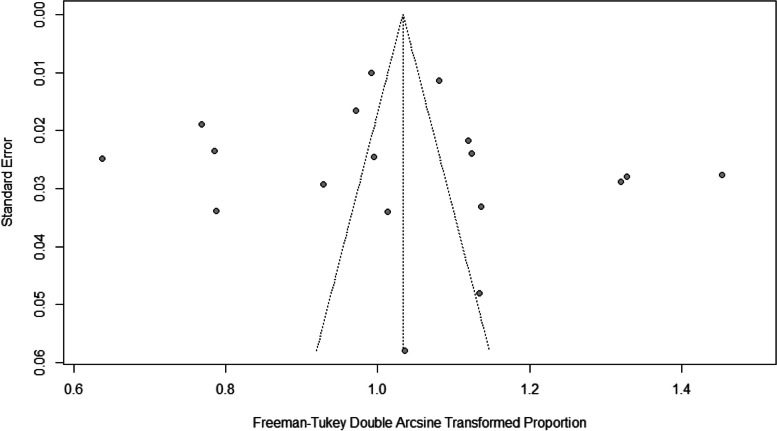
Funnel plot and Egger's test illustrate the publication bias of the included studies

The included cross-sectional studies were characterized by their high level of quality, which was assessed using the JBI-MAStARI tool (Table 1). In the analyses aimed at assessing the prevalence of CVS during the COVID-19 pandemic, it was observed that when applying Egger's test to assess publication bias, a value of *p* = 0.7046 (t = 0.39, df = 16) was obtained. This result suggests that the null hypothesis of symmetry is accepted, indicating that there is no evidence of publication bias in the studies examined (Fig. 2).

### Pooled prevalence of computer vision syndrome

**Fig. 3:**
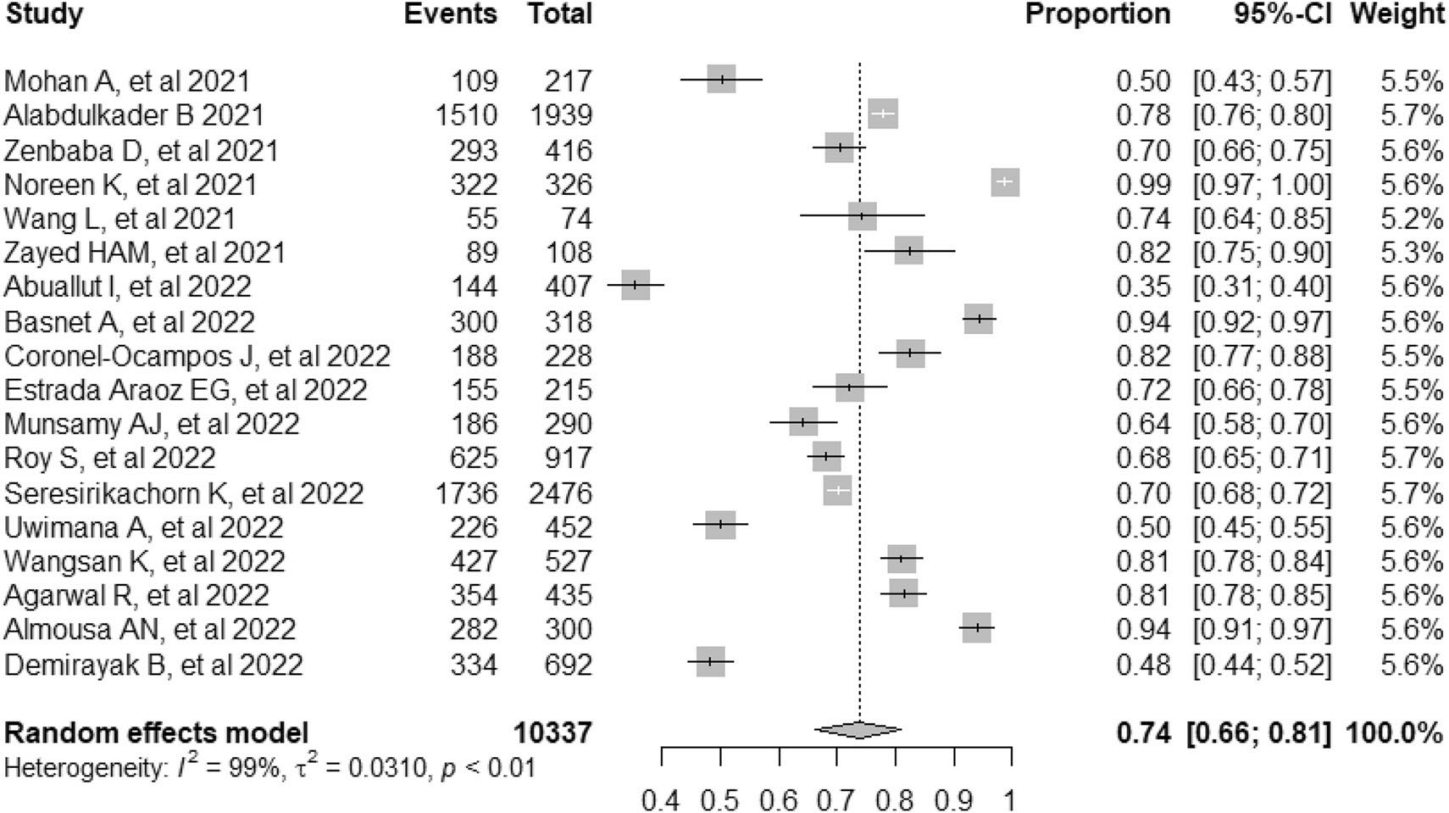
Forest plot showing the pooled prevalence of computer vision syndrome during the COVID-19 pandemic

The pooled prevalence of CVS was 74% (95% CI: 66, 81) [13, 17–33]. The study with the lowest proportion was conducted in Saudi Arabia, with a prevalence of 35% (95% CI: 31, 40) [23], while the highest was conducted in Pakistan, with a prevalence of 99% (95% CI: 97, 100) [20]. The *I*^*2*^ test showed that there was heterogeneity among the included studies (*I*^*2*^ = 99%, *p*-value < 0.01) (Fig. 3) [13, 17–33].

### Subgroup analysis by country

**Fig. 4:**
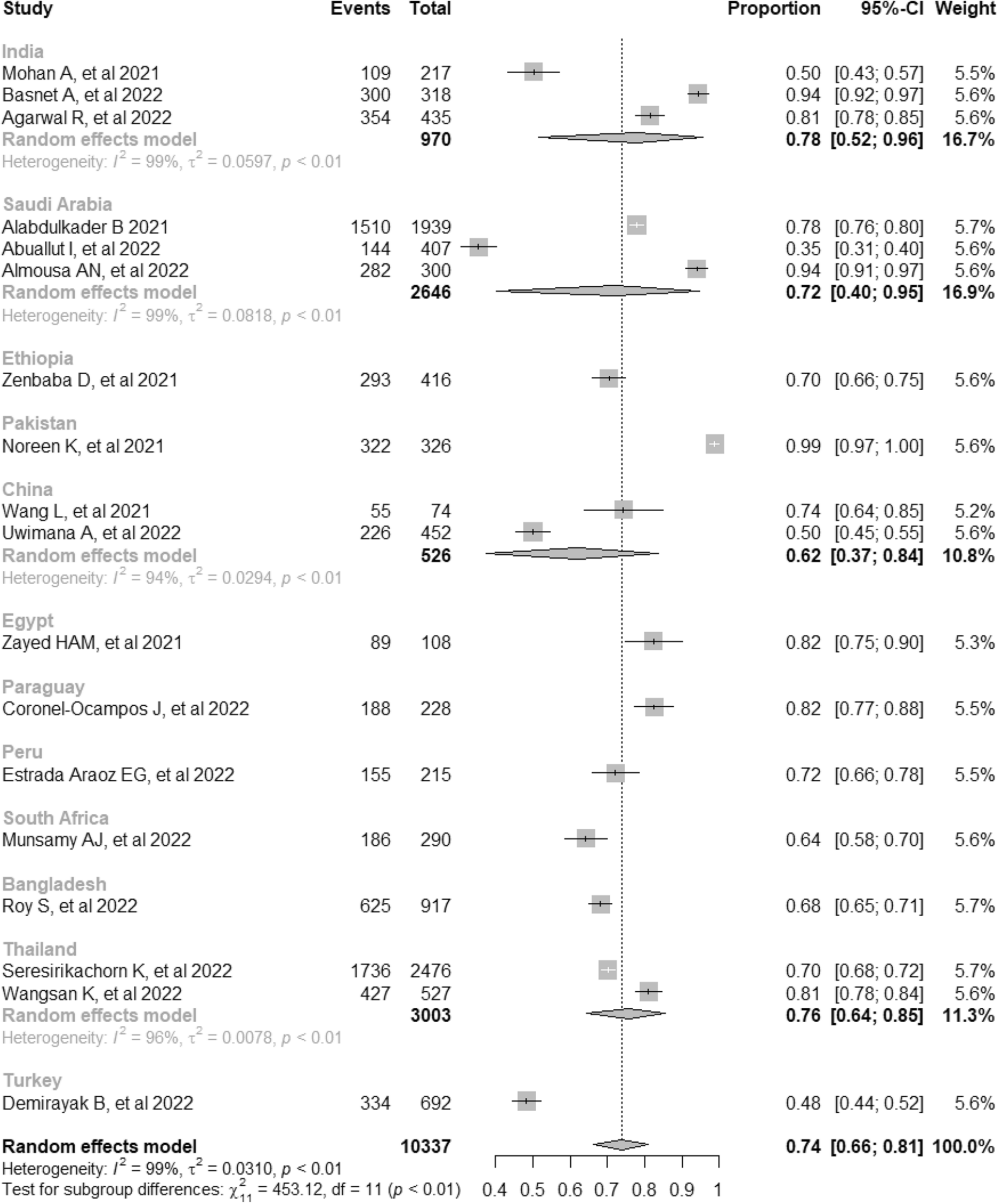
Subgroup analysis by country on computer vision syndrome during the COVID-19 pandemic

Subgroup analysis was performed based on country, and the prevalence of CVS was highest in Pakistan (99%, 95% CI: 97, 100) [20] and lowest in Turkey (48%, 95% CI: 44, 52) [33]. The studies that showed significant heterogeneity were studies in India (*I*^*2*^ = 99%, *p*-value < 0.01), Saudi Arabia (*I*^*2*^ = 99%, *p*-value < 0.01), China (*I*^*2*^ = 94%, *p*-value < 0.01), and Thailand (*I*^*2*^ = 96%, *p*-value < 0.01) (Fig. 4).

### Subgroup analysis by study subjects

**Fig. 5:**
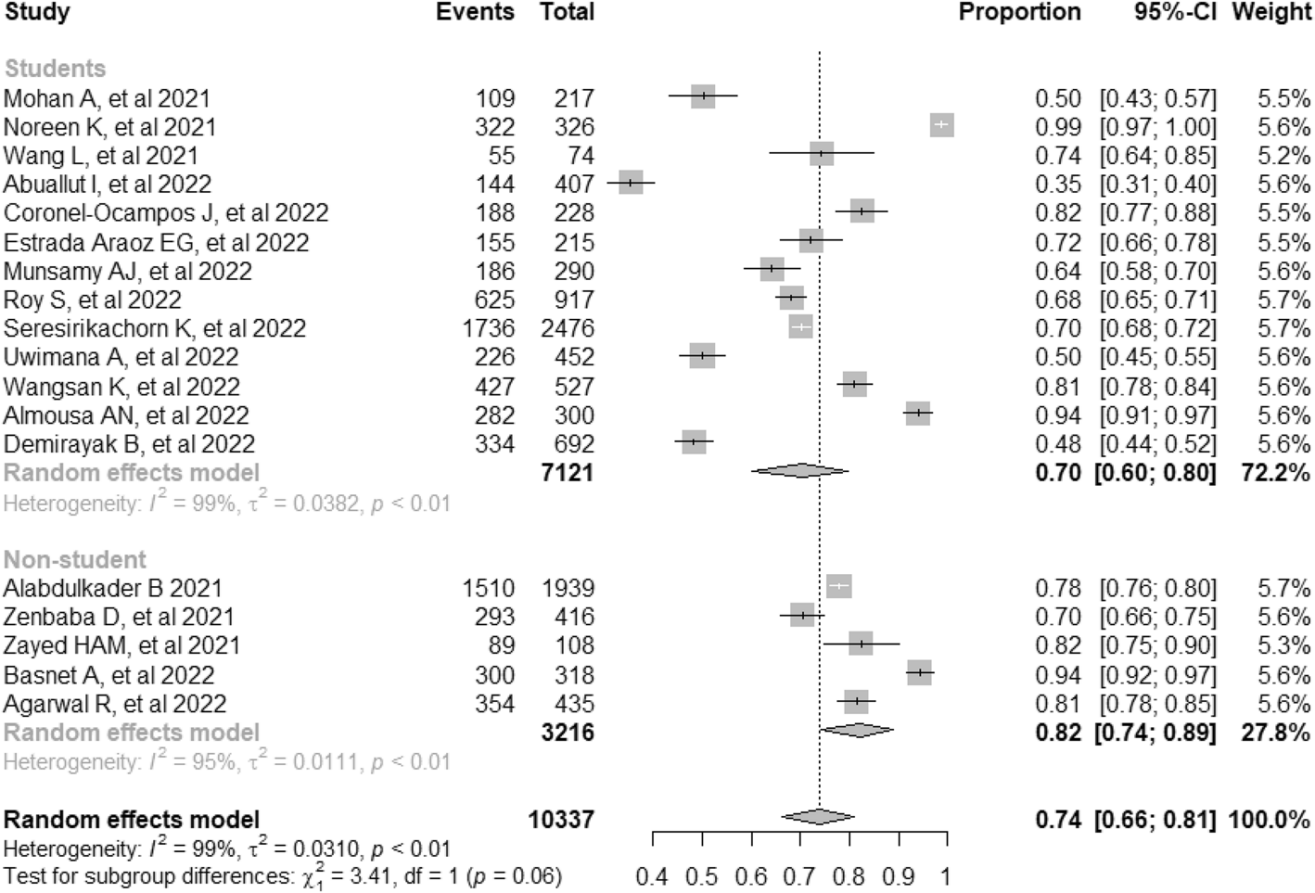
Subgroup analysis by study subjects on computer vision syndrome during the COVID-19 pandemic

A subgroup analysis was performed according to the study subjects, which revealed a prevalence of 82% (95% CI: 74, 89) for computer vision syndrome in non-students [18, 19, 22, 24, 31] and 70% (95% CI: 60, 80) among students [13, 17, 20, 21, 23, 25–30, 32, 33]. However, heterogeneity was observed among the studies included in both groups for both non-student and student subjects (*I*^*2*^ = 95%, *p*-value < 0.01; *I*^*2*^ = 99%, *p*-value < 0.01) (Fig. 5).

## Discussion

Since the onset of the COVID-19 pandemic, numerous countries around the world have implemented restrictions on physical activity and mobility as part of their social isolation and quarantine measures. These measures have had a significant impact on daily life, transforming the reality in which interactions have become predominantly virtual, whether for work, education, shopping, or other activities [34]. Several studies have shown a reduction in physical activity and an increase in sedentary time during the period of confinement due to the COVID-19 pandemic [35–38].

Therefore, the present study aimed to determine the prevalence of CVS during the COVID-19 pandemic, considering the increase in the use of electronic devices and the change in lifestyle towards greater virtualization in areas such as work, education, communications, and various daily activities [39]. Our meta-analysis reveals that during the COVID-19 pandemic, the overall prevalence of CVS increased by 74%, significantly exceeding the overall prevalence estimated in a recent systematic review by Anbesu EW et al. of 66% [3]. However, these results are consistent with the high prevalence rate of CVS in Ethiopia, which reaches 73.21%, as reported in a systematic review conducted by Adane et al. [3, 40]. That difference in prevalence between our study and the other one performed by Anbesu et al. is consistent with the shift in lifestyle during the period of COVID-19 to be more virtual and with the increase in the usage of electronic devices for multiple needs [39, 41]. In addition, several studies have been conducted to assess the impact of digital device usage during the COVID-19 pandemic on eye health, such as the study conducted by Bahkir et al., which found an increase in screen time, and 95% of the participants experienced at least one symptom of digital device use, such as dry eye, eye pain, eye redness, headache, double vision, and others [42]. Furthermore, another study conducted by Alabdulkader et al. reported that the incidence of digital eye strain was 78%, which is positively correlated with duration and the number of devices that are used [18], aside from another study conducted by Usgaonkar et al., which found that 89% of the participants were spending most of the time on social media using electronic devices. Hence, they experienced symptoms such as dry eyes, headaches, and back pain [14].

These results suggest that CVS significantly affected a large portion of the population during this period of health crisis. This underscores the importance of understanding and addressing the potential negative effects of prolonged exposure to electronic device screens.

The sub-group analysis based on the country revealed different prevalence estimates based on the number of studies available per country and justifying the inclusion criteria. The prevalence of CVS in India was estimated at 78%; similar results indicated a prevalence of digital eyestrain in the pre-isolation period of 64.3% [43]. That indicates the impact of increased digital device usage during the lockdown period. Our subgroup analysis revealed a CVS prevalence in Saudi Arabia of 72% based on the three included studies. However, this estimate differs from one setting to another. It varies based on the target population, as CVS prevalence was estimated at 43.5% in another Al Subaie et al. study among the Al-Ahsa population. Besides, another study conducted among radiologists revealed an overall prevalence of 65.4% [44, 45]. Our sub-group analysis revealed that the highest prevalence of CVS was in Pakistan (99%) based on only one included study, and the lowest prevalence was in Turkey (48%) based on only one study. This significant difference between them is based on the difference in the population's culture, behavior, and habits [46, 47].

It was estimated that the prevalence of CVS among students was 70%, which is consistent with their increased usage of computers and learning tools during the COVID-19 lockdown as most educational institutions and universities transformed their learning to be more virtual during the pandemic [48–50].

Our results revealed the variation in the CVS prevalence from one country to another, as shown in the sub-group analysis, and besides other studies conducted, such as the study conducted by Ranasinghe et al. among Sri Lankan computer workers, which reported a CVS prevalence of 67.4%, a survey conducted among the instructors in Ethiopia (70.4%), and a study conducted among undergraduate students in Pakistan (90.5%) [19, 51, 52]. The difference in the prevalence estimates may be attributed to the differences in the study setting, study period, socioeconomic characteristics, awareness of the target population about the preventive measures of using computers and digital devices, and the tool used in the estimation of the prevalence, which mainly depends on subjective questions and differs from one study to another.

Subgroup analysis revealed that the prevalence of CVS was higher in the non-student group (82%), compared to the student group (70%). These results can be attributed to the face-to-face restrictions that the COVID-19 pandemic imposed on educational and work activities, generating a massive transition to virtual environments. This shift resulted in a significant increase in time spent using electronic devices, such as computers, tablets, and cell phones. The non-student population, who were employed in jobs that did not require regular use of electronic devices or who lacked access to formal educational resources, possibly experienced greater exposure to digital screens without the same guidance and eye protection measures. This lack of guidance could have contributed to the higher prevalence of CVS in this group. In contrast, the student population may have demonstrated greater awareness of and access to eye protection measures, such as regular breaks, ergonomic adjustments, and the use of blue light filters. This is due to the guidance provided by educational institutions and the increased attention paid to eye health in the educational environment [53, 54].

The increased prevalence of CVS indicates poor education about this syndrome and the proper ways of using computers and digital devices. Therefore, educational programs should be designed for the general population to increase their awareness of CVS symptoms, the risk factors of this syndrome, the different causes of it, and safety measures to prevent it [55]. We should also promote research to identify the prevalence of CVS in other countries lacking data to have a more precise estimate of the prevalence of this syndrome globally.

This is the first meta-analysis conducted to describe the prevalence of CVS in the context of the COVID-19 pandemic. The results obtained in this study will provide a solid basis on which policymakers can design new policies and follow-up programs to address this syndrome. In addition, this analysis contributed significantly to the identification of gaps in research on the prevalence of CVS, which, in turn, will help guide future measures aimed at preventing its occurrence. Importantly, this study focused on the negative impact on ocular health due to the COVID-19 pandemic. It is important to mention that the present work was carried out following PRISMA guidelines. However, it is relevant to recognize certain limitations in our research. In particular, the limited availability of studies on the prevalence of CVS in a small number of countries limited our analysis. Furthermore, given that the included studies were cross-sectional in nature, we cannot establish a definitive causal relationship. Finally, it is important to keep in mind that our investigation was based on data collected in individual studies during a specific period, implying that circumstances might have changed over time***.***

## Conclusions

This study discovered a high prevalence of computer vision syndrome, affecting 74% of the participants. These results suggest that many individuals may experience prolonged electronic screen usage symptoms, including visual fatigue, headaches or neck pain, dry eyes, and blurred vision, among others. Therefore, it is crucial to emphasize the importance of implementing preventive and therapeutic measures to reduce the risk of developing CVS and improve the affected individuals' quality of life.

## Data Availability

All data generated or analyzed during this study are included in this published article.
